# Early diagnosis and developmental outcome prediction of agenesis of the corpus callosum via an interpretable deep multimodal fusion model

**DOI:** 10.3389/fnins.2026.1812374

**Published:** 2026-04-15

**Authors:** Jing Chen, Wen-Han Zhang, Yang Bai, Lian-Ting Hu, Yu-Yang He, Jing-Jing Li, Nian Zhang, Na Su, Zhi-Sheng Liu, Hong-Min Zhu

**Affiliations:** 1 Department of Rehabilitation Medicine, Wuhan Children's Hospital, Tongji Medical College, Huazhong University of Science and Technology, Wuhan, China; 2 Department of Radiology, Wuhan Children’s Hospital (Wuhan Maternal and Child Healthcare Hospital), Tongji Medical College, Huazhong University of Science & Technology, Wuhan, China; 3 National Engineering Research Center for Multimedia Software, School of Computer Science, Wuhan University, Wuhan, China; 4 Data Center, Wuhan Children's Hospital (Wuhan Maternal and Child Healthcare Hospital), Tongji Medical College, Huazhong University of Science and Technology, Wuhan, China; 5 Department of Neurology, Wuhan Children's Hospital, Tongji Medical College, Huazhong University of Science and Technology, Wuhan, China

**Keywords:** agenesis of the corpus callosum, deep neural network, interpretable machine learning, neurodevelopmental outcome, SHAP

## Abstract

**Objective:**

Agenesis of the corpus callosum (ACC) presents with highly heterogeneous clinical features. Common methods rarely achieve accurate prenatal or early postnatal diagnosis and prognosis. We aimed to develop and test an interpretable deep neural network (DNN) that combines multimodal clinical data to improve diagnostic accuracy and neurodevelopmental outcome prediction.

**Methods:**

We collected data from 205 pediatric patients with ACC at Wuhan Children’s Hospital between 2016 and 2024. A total of 27 clinical features were extracted, including neuroimaging findings, perinatal risk factors, and follow-up developmental quotients (Gesell Developmental Schedules and Gross Motor Function scores). Five-fold cross-validation was adopted. We built an eight-layer fully connected DNN with ReLU activation in the hidden layers. For categorical endpoints, a sigmoid output layer with binary cross-entropy loss was used. For continuous endpoints, a linear output layer with mean squared error loss was used. SHAP (Shapley Additive Explanations) values were used to quantify the contribution of individual features to model predictions. Performance was compared with a support vector machine (SVM) baseline and across hyperparameter settings. Area under the receiver-operating-characteristic curve (AUC), F1 score, precision, recall, mean absolute error (MAE), mean squared error (MSE), and coefficient of determination (R^2^) served as primary metrics.

**Results:**

Across 12 neurodevelopmental disorders, the model reached an average AUC of 0.97. AUCs for intellectual disability, autism spectrum disorder (ASD), attention deficit hyperactivity disorder (ADHD), specific learning disorder and developmental coordination disorder ranged from 0.98 to 1.00. Prediction remained moderate for cerebral palsy (AUC = 0.74) and epilepsy (AUC = 0.67). MAE for both Gesell and Gross Motor Function scores was 0.10, with corresponding R^2^ values of 0.62 and 0.63. SHAP analysis identified extracranial malformation (clinical type III), facial dysmorphism and birth weight as the most influential features for developmental outcome. The DNN model outperformed the SVM baseline, with an AUC improvement of 0.16 for communication disorder and an R^2^ increase of 0.19 for Gesell score (*p* < 0.001). Ablation experiments confirmed eight layers, sixteen neurons per layer, a learning rate of 0.01 and ten training epochs as the optimal configuration. Additional layers or higher learning rates caused overfitting.

**Conclusion:**

The proposed interpretable DNN framework outperforms traditional classifiers in early ACC diagnosis and developmental outcome prediction. It provides a potential tool for clinical decision support. Larger samples and integration of raw imaging data are needed to enhance prediction of complex phenotypes such as cerebral palsy and epilepsy.

## 1 Introduction

Agenesis of the corpus callosum (ACC) is one of the most common congenital brain malformations. Its prevalence ranges from 0.3–0.7% in the general population and 3–5% in neurodevelopmental disorders (Stoll et al., 2019; Bayram et al., 2020). The primary characteristic of ACC is partial or complete absence of the corpus callosum, which serves as a crucial bundle of nerve fibers connecting the left and right cerebral hemispheres (Rotmensch and Monteagudo, 2020). The clinical presentation of ACC shows high variability, ranging from asymptomatic to severe neurodevelopmental disorders. Even among patients with the same ACC subtype, prognosis varies significantly. Common clinical features include reduced transmission of sensorimotor information, increased cognitive processing times, and impaired ability to process complex information or tasks (Wright and Booth, 2023). The prevalence of neurodevelopmental disorders varies significantly, with approximately 35–70% of cases presenting intellectual disability, 20–50% with epilepsy, and 13–40% with cerebral palsy or movement disorders (Wright and Booth, 2023; Jańczewska et al., 2023; Raile et al., 2020; Pânzaru et al., 2022). Given the substantial variability in clinical outcomes, achieving early and accurate prognostic assessment remains a major challenge in pediatric neurology and developmental medicine.

Despite recent advances in neuroimaging and genetic diagnostic technologies, predicting developmental outcomes in children with ACC remains clinically challenging. Traditional prognostic models for ACC mainly rely on single clinical indicators, such as imaging findings or gestational data (Jańczewska et al., 2023; Raile et al., 2020). Furthermore, existing research primarily focuses on etiological categorical prediction tasks and diagnostic classification of the disease (Pânzaru et al., 2022), rather than predicting long-term functional outcomes. This limits their practical value in clinical decision-making and early intervention.

In recent years, machine learning (ML) and deep learning (DL) approaches have shown promise in modeling complex, nonlinear relationships within high-dimensional datasets (Rahman et al., 2024; Dayarathna et al., 2024; Rana and Bhushan, 2022). These techniques have been increasingly applied in pediatric neuroimaging and neurodevelopmental research, demonstrating superior performance over conventional statistical methods (Zhao et al., 2025). However, DL applications specifically targeting ACC remain limited, particularly in the pediatric population. Furthermore, few studies have explored the integration of multimodal data, including neuroimaging findings, perinatal risk factors, and early developmental assessments to predict outcomes in ACC.

Multimodal data fusion combines complementary information from different data sources to improve model performance. It has become a key strategy in medical predictive modeling tasks (Zhang et al., 2020; Bi et al., 2019). For ACC, integrating structured neuroimaging features with clinical and demographic variables may provide a more comprehensive representation of individual risk profiles and enhance the accuracy of outcome prediction. Nevertheless, the optimal methods for fusing such heterogeneous data and interpreting model predictions in a clinically meaningful way remain underexplored.

In this study, we develop and validate a deep learning–based framework that integrates multimodal clinical data to predict developmental outcomes in children with ACC. We use a retrospective cohort of 205 pediatric patients treated at Wuhan Children’s Hospital between 2016 and 2024. To construct a high-quality modeling dataset, we performed systematic data preprocessing including tailored imputation strategies for mixed-type variables, one-hot encoding and scaling procedures, and correlation-based feature selection to ensure data integrity and optimize feature relevance. We then combine neuroimaging indicators, prenatal and perinatal risk factors, and early developmental assessments to train and evaluate predictive models. To capture the complex non-linear interactions among clinical factors, we design and optimize a deep neural network architecture capable of predicting both categorical and continuous developmental outcomes. Model performance is rigorously assessed using cross-validation, task-specific loss functions, and multiple evaluation metrics. In addition, we employ SHAP (Shapley Additive explanations) to quantify the contribution of individual clinical features, which enhanced model interpretability and identifies key predictors of developmental risk.

## 2 Methods

In this paper, we used artificial intelligence (AI) techniques, particularly deep learning (Rasmy et al., 2021; Esteva et al., 2019), to provide a transformative solution. This approach enables the analysis of large multi-dimensional datasets to enhance the accuracy of diagnostic and prognostic predictions for ACC.

The methodological framework of this paper is structured into two main phases: data preparation and predictive modeling. In the data preparation phase, we focused on data collection and data preprocessing. This phase ensured data integrity, consistency, and readiness for subsequent modeling. The predictive modeling phase involves model architecture design, training and evaluation procedures. By integrating these two phases, the proposed framework offers a comprehensive approach for analyzing and predicting developmental outcomes and prognostic indicators in patients with ACC.

## 3 Data preparation

### 3.1 Data collection

This study was approved by the Ethics Committee of Wuhan Children’s Hospital (Grant No. 2022R043-E01). We retrospectively collected the records of in-patients diagnosed with corpus callosum dysplasia at Wuhan Children’s Hospital between August 2016 and August 2024.

In this phase, the collected data were preprocessed to prepare for the subsequent modeling. This preprocessing included handling missing values, feature scaling and feature selection, ensuring that the data are presented in an organized and standardized format, ready for downstream modeling. The following variables were extracted: age at first admission, birth weight, gestational age, mode of delivery, maternal history, past medical history, family history, physical examination findings, results of genetic testing, cranial magnetic resonance imaging (MRI) studies, electroencephalograms, Gesell Developmental Schedules (a standardized and widely used developmental diagnostic scale for children aged 0–6 years, evaluating five major domains: adaptive behavior, gross motor skills, fine motor skills, language, and personal-social skills; the primary score used as a continuous outcome was the Developmental Quotient [DQ]), Gross Motor Function Measure (GMFM)scores, and ancillary investigations (echocardiography, abdominal ultrasonography of liver and spleen, genital/reproductive and urinary-tract ultrasonography, chest CT or radiography, etc.).

Inclusion criterion: MRI-based radiological diagnosis of corpus callosum dysplasia (complete or partial agenesis, hypoplasia). Exclusion criteria included: (1) unavailable or non-diagnostic MRI; (2) insufficient clinical documentation; (3) acquired callosal abnormalities (e.g., post-traumatic changes).

From the radiology reports, structured labels were extracted, which included: ACC type (complete/partial/hypoplasia), presence of associated intracranial malformations (e.g., cortical dysplasia, heterotopia, cerebellar abnormalities, hydrocephalus), and any noted extracranial malformations. These findings were categorized into the ‘Clinical type’ features (I: isolated ACC; II: ACC with intracranial malformations; III: ACC with extracranial malformations) used in subsequent modeling.

**Figure 1:**
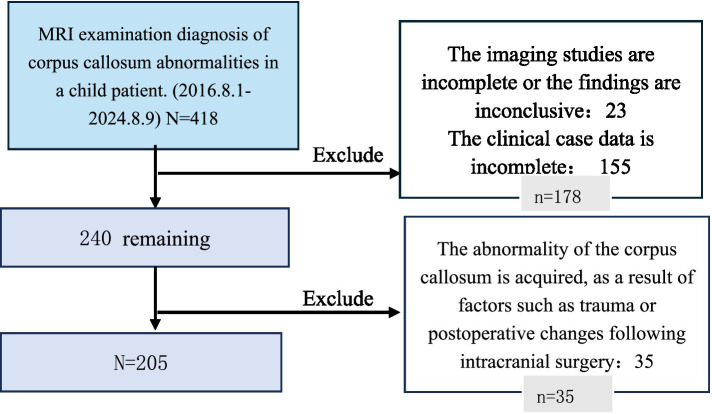
Inclusion and exclusion diagram. Flowchart showing the patient selection process for the study cohort. A total of 312 patients with corpus callosum dysplasia were initially screened from the hospital’s big data platform between August 2016 and August 2024. After applying inclusion and exclusion criteria, 205 patients were included in the final analysis. ACC, agenesis of the corpus callosum; MRI, magnetic resonance imaging.

The patient selection process is detailed in Figure 1. Ultimately, 205 subjects were included in this study. Their demographic and clinical characteristics are summarized in Table 1.

**Table 1:** Demographic and clinical characteristics of subjects with agenesis of the corpus callosum (ACC) by clinical type.

Characteristic	Clinical type I of ACC			χ^2^/H	*p* value	Clinical type II of ACC		χ^2^/H	*p* value	Clinical type III of ACC		χ^2^/H	*p* value
	Complete ACC	Partial ACC	CCH			Isolated type	Complex type			Non-syndromic	Syndromic		
Total	4 (1.9%)	42 (20.5%)	159 (77.6%)	—	—	40 (19.5%)	165 (80.5%)	—	—	99 (48.3%)	106 (51.7%)	—	—
Gender													
Male	3 (75.0%)	24 (57.1%)	102 (64.2%)	0.88	0.645	28 (66.7%)	100 (60.6%)	2.83	0.243	60 (60.6%)	68 (64.2%)	0.11	0.744
Female	1 (25.0%)	18 (42.9%)	57 (35.8%)			12 (33.3%)	65 (39.4%)			39 (39.4%)	38 (35.8%)		
Age (Months)													
Median	12.5	24	15	2.65	0.265	12	20	5.05	0.080	23	14	5.53	**0.019**
Birth weight													
Low birth weight	1 (25.0%)	8 (19.0%)	71 (44.7%)	11.04	**0.026**	10 (25.0%)	70 (42.4%)	5.68	0.224	36 (36.4%)	44 (41.5%)	0.89	0.639
Normal birth weight	3 (75.0%)	34 (81.0%)	85 (53.5%)			29 (72.5%)	93 (56.4%)			61 (61.6%)	61 (57.6%)		
Large birth weight	0 (0.0%)	0 (0.0%)	3 (1.8%)			1 (2.5%)	2 (1.2%)			2 (2.0%)	1 (0.9%)		
Gestational age													
preterm birth	1 (25.0%)	5 (11.9%)	67 (41.1%)	13.45	**<0.005**	6 (15.0%)	67 (40.6%)	10.75	**<0.005**	35 (35.4%)	38 (35.8%)	0.000	1.000
term birth	3 (75.0%)	37 (88.1%)	92 (57.9%)			34 (85.0%)	98 (59.4%)			64 (64.6%)	68(64.2%)		
Perinatal asphyxia													
Yes	1 (25.0%)	14 (33.3%)	71 (44.7%)	2.60	0.627	11 (27.5%)	75 (45.5%)	10.13	**0.038**	42 (42.4%)	44 (41.5%)	0.97	0.615
No	3 (75.0%)	28 (66.7%)	88 (55.3%)			29 (72.5%)	90 (54.5%)			57 (57.6%)	62(58.5%)		
Neonatal pathological jaundice													
Yes	1 (25.0%)	9 (21.4%)	40 (25.2%)	0.29	0.864	13 (32.5%)	37 (22.4%)	1.99	0.369	18 (18.2%)	32(30.2%)	3.53	0.061
No	3 (75.0%)	33 (78.6%)	119 (74.8%)			27 (67.5%)	128 (77.6%)			81 (81.8%)	74(69.8%)		
Intellectual disability													
Yes	2 (50.0%)	40 (95.2%)	140 (88.1%)	7.85	**0.020**	32 (80.0%)	150 (90.9%)	3.87	0.144	85 (85.9%)	97(91.5%)	1.07	0.300
No	2 (50.0%)	2 (4.8%)	19 (11.9%)			8 (20.0%)	15 (9.1%)			14 (14.1%)	9(8.5%)		
Motor disorder													
Yes	3 (75.0%)	39 (92.9%)	154 (96.9%)	5.30	0.071	39 (97.5%)	157 (95.2%)	0.49	0.784	93 (93.9%)	103(97.2%)	0.60	0.440
No	1 (25.0%)	3 (7.1%)	5 (3.1%)			1 (2.5%)	8 (4.8%)			6 (6.1%)	3(2.8%)		
Language disorder													
Yes	2 (50.0%)	38 (90.5%)	126 (79.2%)	5.31	0.070	27 (67.5%)	139 (84.2%)	5.93	0.052	81 (81.8%)	85(80.2%)	0.02	0.879
No	2 (50.0%)	4 (9.5%)	33 (20.8%)			13 (32.5%)	26 (15.8%)			18 (18.2%)	21(19.8%)		

ACC = agenesis of the corpus callosum; CCH=Hypoplasia of the corpus callosum. Data are presented as n (%) or median as appropriate. Statistical comparisons were performed using chi-square test or Kruskal–Wallis test as appropriate. *p* values < 0.05 are considered statistically significant and are shown in bold. χ^2^ = chi-square test for categorical variables; H = Kruskal-Wallis H test for continuous variables (Age). Age at first admission/assessment when baseline clinical and imaging data were collected for model input, while the neurodevelopmental diagnoses were determined based on longitudinal follow-up evaluations conducted approximately 2 years after the initial admission age.

The neurodevelopmental disorder diagnoses used as prediction targets were established during longitudinal follow-up (detailed in the Table 1 footnote), not at initial admission.

### 3.2 Data preprocessing

#### 3.2.1 Missing values

Missing values pose a common challenge in the preprocessing phase of predictive modeling (Yoon et al., 2018; Che et al., 2018), disrupting the learning process and decreasing model accuracy by hindering deep learning algorithms’ ability to capture underlying patterns. In our dataset, missing values are mainly represented as NaN, necessitating careful handling to ensure the reliability of modeling outcomes.

The direct removal of records with missing values was deemed inappropriate, as such an approach would reduce the size of the training data and consequently limit the model’s generalization capacity for data with missing values in real-world applications. To address this challenge, we implement a tailored imputation strategy based on the different types of variables:

1. Categorical variables: for categorical variables (e.g., clinical type of ACC, delivery category and birth asphyxia, and genetic testing results), missing values were imputed with a placeholder category (−1) to explicitly identify those entries, allowing the model to handle them appropriately.
2. Numerical variables: for numerical variables, such as birth weight, missing values were replaced by the mean value of the variable. This approach ensures that imputation preserves the distribution of the numerical features while maintaining their statistical integrity. For the Gesell Developmental Quotient (DQ), seven children had missing values due to the Gesell scale being inapplicable for their age at the follow-up time point; these missing values were imputed using the mean DQ score from the entire dataset.

The above imputation strategies were designed to address two critical challenges: ensuring that the model utilizes as much information as possible from the dataset, while minimizing biases introduced by the imputation process. By tailoring imputation strategies, this approach enhances the dataset’s completeness and supports the development of more accurate predictive models.

#### 3.2.2 Feature scaling

Feature scaling is a crucial step in data preprocessing, particularly when working with deep learning algorithms that are sensitive to the magnitude of features (Shao et al., 2020; Rashmi and Shantala, 2024). Scaling ensures that all features contribute equally to the learning process, preventing variables with larger scales from dominating those with smaller scales. In this study, feature scaling was applied differently to categorical and numerical variables, as described below:

1. Categorical variables: categorical variables were converted into a numerical format using one-hot encoding, ensuring that each category was represented independently without imposing any ordinal relationship.
2. Numerical variables: for numerical variables, normalization (also known as min-max scaling) and standardization (z-score scaling) were applied to bring all features to a comparable scale, thereby eliminating the influence of different magnitudes.
  - Normalization: this scales the feature values to a range of [0,1] using the following formula Equation 1:

$$X_{\mathrm{norm}}=\frac{X-X_{\min}}{X_{\max}-X_{\min}}$$ (1)

- Standardization: this transforms the feature values to have a mean of 0 and a standard deviation of 1 using the following formula Equation 2:

$$\begin{array}{c} X_{\mathrm{std}}=\frac{X-\mu }{\sigma } \end{array}$$ (2)

where μ is the mean and σ is the standard deviation of the feature.

#### 3.2.3 Feature selection

**Figure 2:**
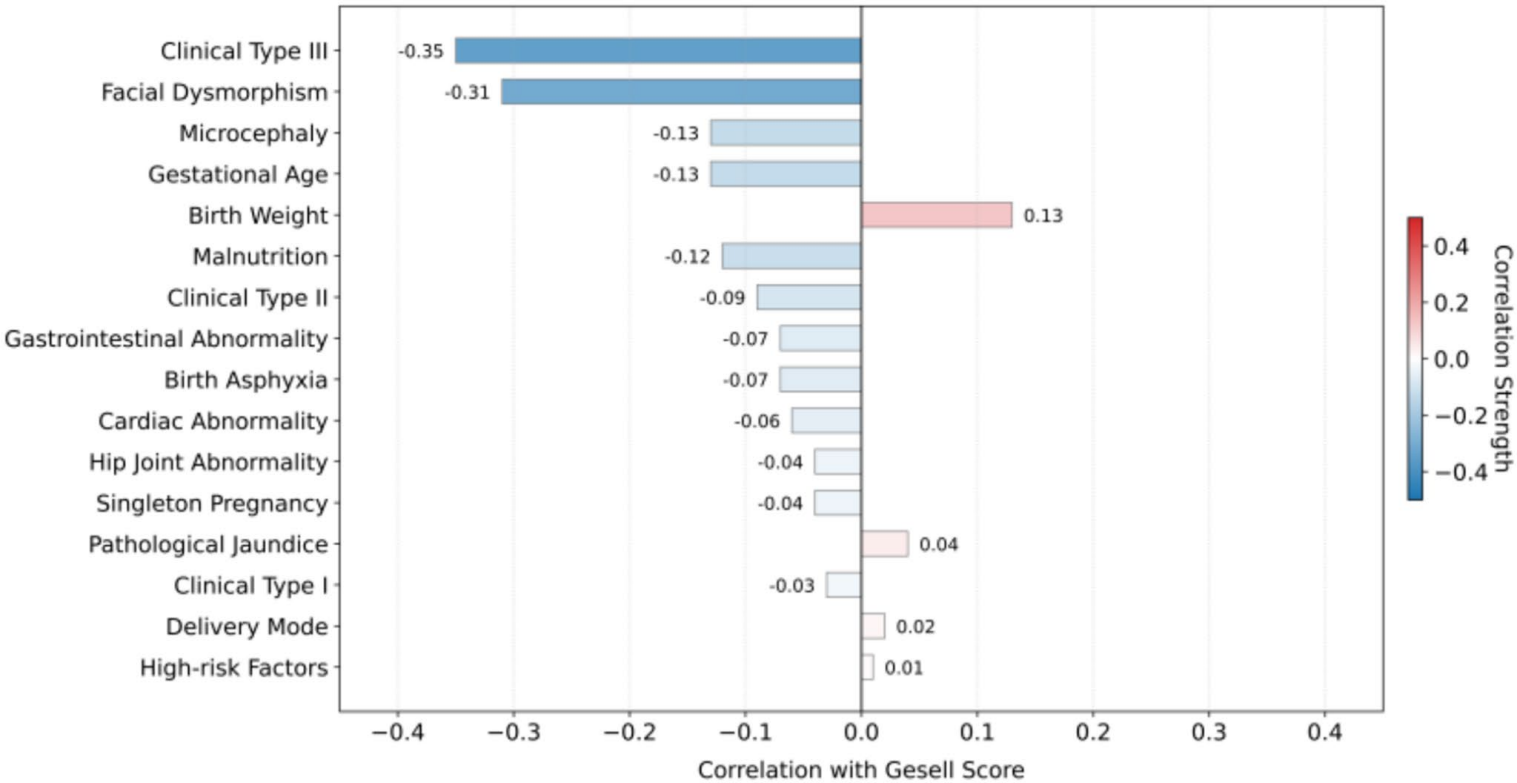
The illustration of correlation coefficient of each input variable with respect to the Gesell score. Heatmap showing the correlation between 27 clinical features and the Gesell developmental score. Point-Biserial Correlation was used for categorical variables and Pearson’s Correlation for numerical variables. Delivery mode and high-risk factors (absolute correlation coefficient ≤ 0.02) were excluded from model training due to negligible correlation with the target variable.

Feature selection plays a critical role in data preprocessing, as it identifies the most relevant features for building a robust and interpretable predictive model. Including irrelevant features may introduce noise into the model, leading to overfitting, where the model learns patterns from the noise rather than the underlying relationships. In this study, Gesell Score is employed as the primary target variable for prediction, and the relevance of each input feature to the Gesell Score is systematically evaluated. To quantify the relationship between input variables and the Gesell Score, given that the input variables consisted of both categorical and numerical types, we employed Point-Biserial Correlation and Pearson’s Correlation to assess their relevance to the Gesell Score (Amirahmadi et al., 2023), respectively. Both correlation coefficients range from −1 to 1, where values closer to 1 or −1 indicate stronger positive or negative associations, while values around 0 suggest little relevance. The overall correlation is illustrated in Figure 2, which provides a comprehensive overview of the relevance between each input variable and the Gesell Score. Based on the reported correlation coefficients, we excluded Delivery Mode and High-risk Factors from the model training, as their coefficients were close to 0 (with absolute values ≤ 0.02), indicating almost no correlation with the predictive variables.

## 4 Predictive modeling

In this ACC diagnostic prediction task, the variables exhibited complex and non-linear interactions, such as dependencies between clinical indicators or symptoms. Traditional linear models struggle to model these complex relationships effectively, while DNNs excel at capturing intricate, non-linear relationships in high-dimensional data (Rajkomar et al., 2018; Rasmy et al., 2021; Klug et al., 2024). DNNs achieve this through their multi-layered architecture, which extracts hierarchical representations of the data (Rong et al., 2025). Each layer learns increasingly abstract features, enabling the model to make accurate predictions.

**Figure 3:**
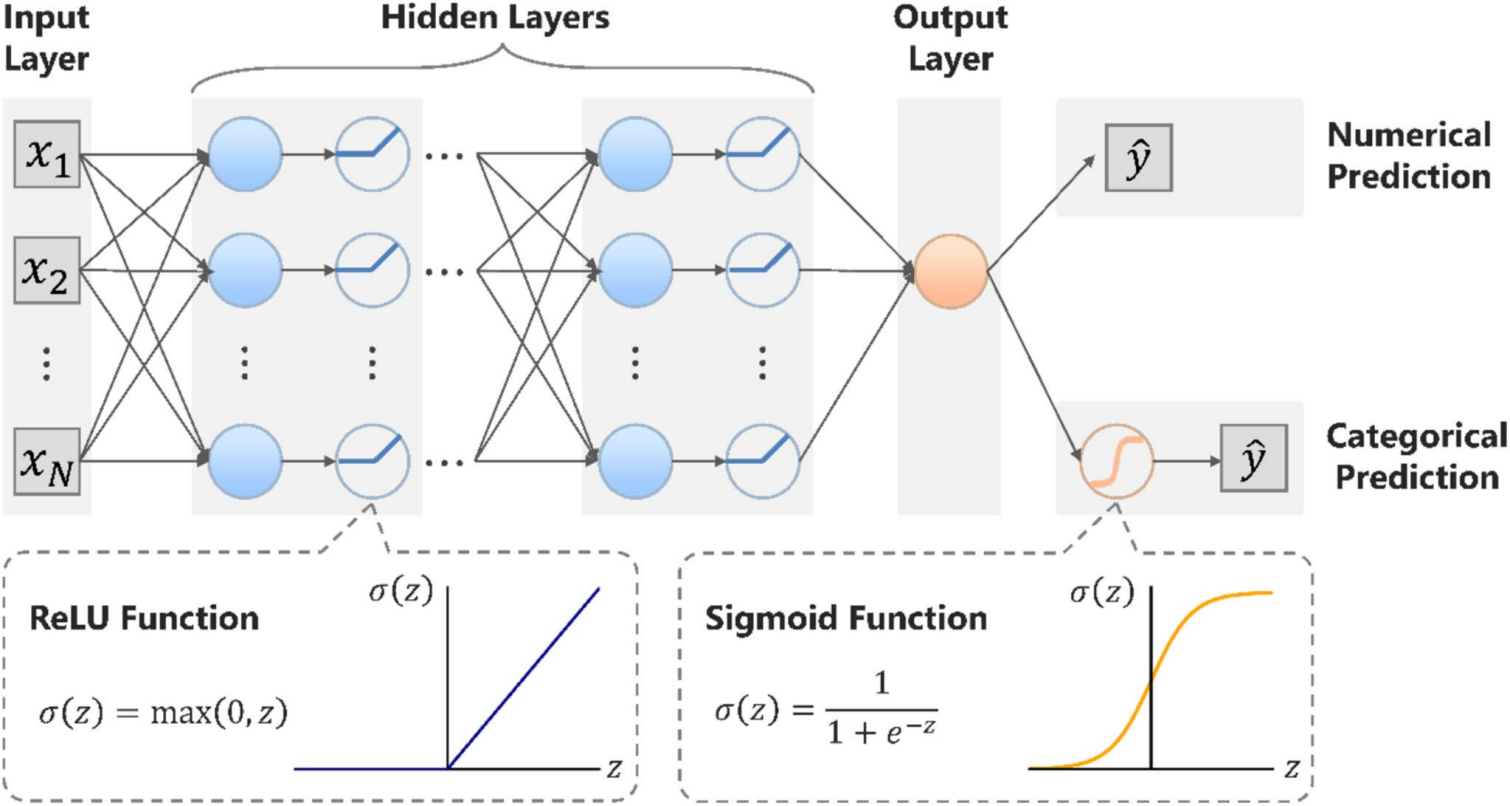
The overall architecture of the ACC prediction model. The model consists of an input layer (27 neurons), eight hidden layers (16 neurons per layer with ReLU activation), and an output layer. Categorical predictions used sigmoid activation with binary cross-entropy loss, while numerical predictions use linear activation with mean squared error loss. ACC, agenesis of the corpus callosum; ReLU, rectified linear unit; BCE, binary cross-entropy; MSE, mean squared error.

Specifically, we denote the training data as $D=\left\{\left(X_{1},y_{1}\right),\left(X_{2},y_{2}\right),\cdots ,\left(X_{N},y_{N}\right)\right\}$ , where $X_{i}\in R^{d}$ represents the feature vector of the i -th sample with dimension d=27 in this paper, including one-hot encoded dimensions, and $y_{i}$ is the corresponding class label. The predictive model consisted of an input layer, multi-layer hidden layers, and an output layer. The overall architecture is shown in Figure 3.

### 4.1 Input layer

The input layer receives the feature vector $X_{i}$ , where each dimension corresponds to a specific input feature, including one-hot encoded variables. The input layer had 27 neurons, one for each dimension.

### 4.2 Hidden layers

The architecture consisted of L hidden layers, each designed to extract hierarchical features from the input data through a combination of linear transformations and non-linear activations. Below, we provide a detailed description of the computations performed in each hidden layer.

Let $h^{\left(l\right)}\in R^{n_{l}}$ denote the output of the l -th hidden layer, where $n_{l}$ is the number of neurons in the l -th layer. The input to the first hidden layer is the feature vector $h^{\left(0\right)}=X$ . For subsequent layers, the input is the output of the previous layer. In the l -th hidden layer, the input $h^{\left(l-1\right)}$ undergoes a linear transformation, defined as Equation 3:


$$\begin{array}{c} z^{\left(l\right)}=W^{\left(l\right)}h^{\left(l-1\right)}+b^{\left(l\right)} \end{array}$$ (3)

where $W^{\left(l\right)}\in R^{n_{l}\times n_{l-1}}$ is the weight matrix of the l -th layer, $b^{\left(l\right)}\in R^{n_{l}}$ is the bias vector of the l -th layer, and $z^{\left(l\right)}\in R^{n_{l}}$ is the intermediate output before applying the activation function. The weights $W^{\left(l\right)}$ and biases $b^{\left(l\right)}$ are learned during training to optimize the model’s predictive performance. Subsequently, the intermediate output $z^{\left(l\right)}$ is passed through the activation function ReLU (Equation 4) to learn complex non-linear relationships in the data:


$$\begin{array}{c} h^{\left(l\right)}=\mathrm{ReLU}\left(z^{\left(l\right)}\right)=\max\left(0{\mathrm{,z}}^{\left(l\right)}\right) \end{array}$$ (4)

where $h^{\left(l\right)}\in R^{n_{l}}$ is the final output of the l -th hidden layer. We chose ReLU as the activation function for its computational efficiency and ability to mitigate the vanishing gradient problem, enabling faster and more stable training.

### 4.3 Output layer

The output layer was designed to perform ACC prediction. It processes the learned features from the hidden layers and maps them to the corresponding output format. Specifically, the output of the last hidden layer $h^{\left(L\right)}$ is fed into the output layer to perform a linear transformation (Equation 5):


$$z=W^{\left(o\right)}h^{\left(L\right)}+b^{\left(o\right)}$$ (5)

where $h^{\left(L\right)}\in R^{n_{L}}$ is the feature vector from the final hidden layer, $W^{\left(o\right)}\in R^{1\times n_{L}}$ is the weight matrix of the output layer to map the hidden features to a single output neuron, $b^{\left(o\right)}\in R$ is the bias term in the output layer, and z∈R is the intermediate output. Depending on the nature of the prediction task, the intermediate output z is transformed using an appropriate activation function to produce the final prediction output $\hat{y}$ .

1. Categorical prediction: for categorical prediction, the intermediate output z is passed through the sigmoid activation function (Equation 6), which makes the predictive output constrained to [0,1], representing the predicted probability of the positive class. The output is mathematically expressed as:

$$\begin{array}{c} \hat{y}=\mathrm{sigmoid}\left(z\right)=\frac{1}{1+e^{-z}} \end{array}$$ (6)

The predicted probability $\hat{y}$ can subsequently be thresholder to classify the input as belonging to the positive or negative class.

1. Numerical prediction: for numerical prediction, no activation function is applied, resulting in a linear output neuron that produces a continuous scalar value. The output for numerical prediction is given by Equation 7:

$$\begin{array}{c} \hat{y}=z \end{array}$$ (7)

This allows the model to generate outputs that span the full range of real numbers, making it suitable for predicting continuous variables.

### 4.4 Loss function

The choice of loss function was determined by the nature of the prediction task. For this framework, we utilized different loss functions to align with the specific requirements of categorical and numerical predictions.

1. Categorical Prediction: for categorical prediction, we employed the binary cross-entropy (BCE) loss (Equation 8) to measure the discrepancy between the predicted probability and the true label:

$$\begin{array}{c} L_{\mathrm{BCE}}=-\frac{1}{N}\overset{N}{\underset{i=1}{\sum }}\left[y_{i}\log{\hat{y}}_{i}+\left(1-y_{i}\right)\log\left(1-{\hat{y}}_{i}\right)\right] \end{array}$$ (8)

where $y_{i}\in \left\{0,1\right\}$ is the ground truth label of i -th sample $X_{i}$ , and ${\hat{y}}_{i}\in \left[0,1\right]$ denotes the predictive output. By minimizing BCE loss, the model was encouraged to generate probabilities that closely matched the ground truth labels, effectively distinguishing between different classes.

1. Numerical prediction: for numerical prediction, the mean squared error (MSE) loss (Equation 9) was utilized to measure the average squared difference between the predicted value and the true target value:

$$\begin{array}{c} L_{\mathrm{MSE}}=\frac{1}{N}\overset{N}{\underset{i=1}{\sum }}{\left({\hat{y}}_{i}-y_{i}\right)}^{2} \end{array}$$ (9)

By minimizing the MSE loss, the model learned to make accurate predictions for continuous numerical variables. This loss function is commonly used in numerical prediction tasks due to its ability to penalize large prediction errors more heavily than smaller ones, encouraging the model to reduce significant deviations from the ground truth.

By adopting these task-specific loss functions, the framework was optimized for both categorical and numerical predictions, ensuring a robust and flexible approach tailored to the diverse nature of ACC-related data.

## 5 Experiments

In this section, we rigorously evaluate the effectiveness of the constructed DNN-based prediction model using the collected dataset. The experimentation is structured to provide a comprehensive analysis of the model’s performance and insights into its predictive capabilities. The following content is organized into four sections: Implementation Details, Validation Methodology, Evaluation Metrics, and Experimental Results and Analysis.

### 5.1 Implementation details

In constructing the predictive model, we designed an architecture comprising L=8 hidden layers, with each layer consisting of $n_{l}=16$ neurons ( l=1,2,⋯,8) . The training process was set with a batch size of 32 to ensure a balanced approach between learning speed and resource utilization. The learning rate was configured at 0.01 , facilitating a steady convergence across 10 training epochs. Stochastic Gradient Descent (SGD) was selected as the optimizer (Bottou, 2012). At the outset of training, all model parameters were randomly initialized. Subsequently, the gradients were then calculated through backpropagation, a crucial method for optimizing model weights iteratively.

### 5.2 Validation process

**Figure 4:**
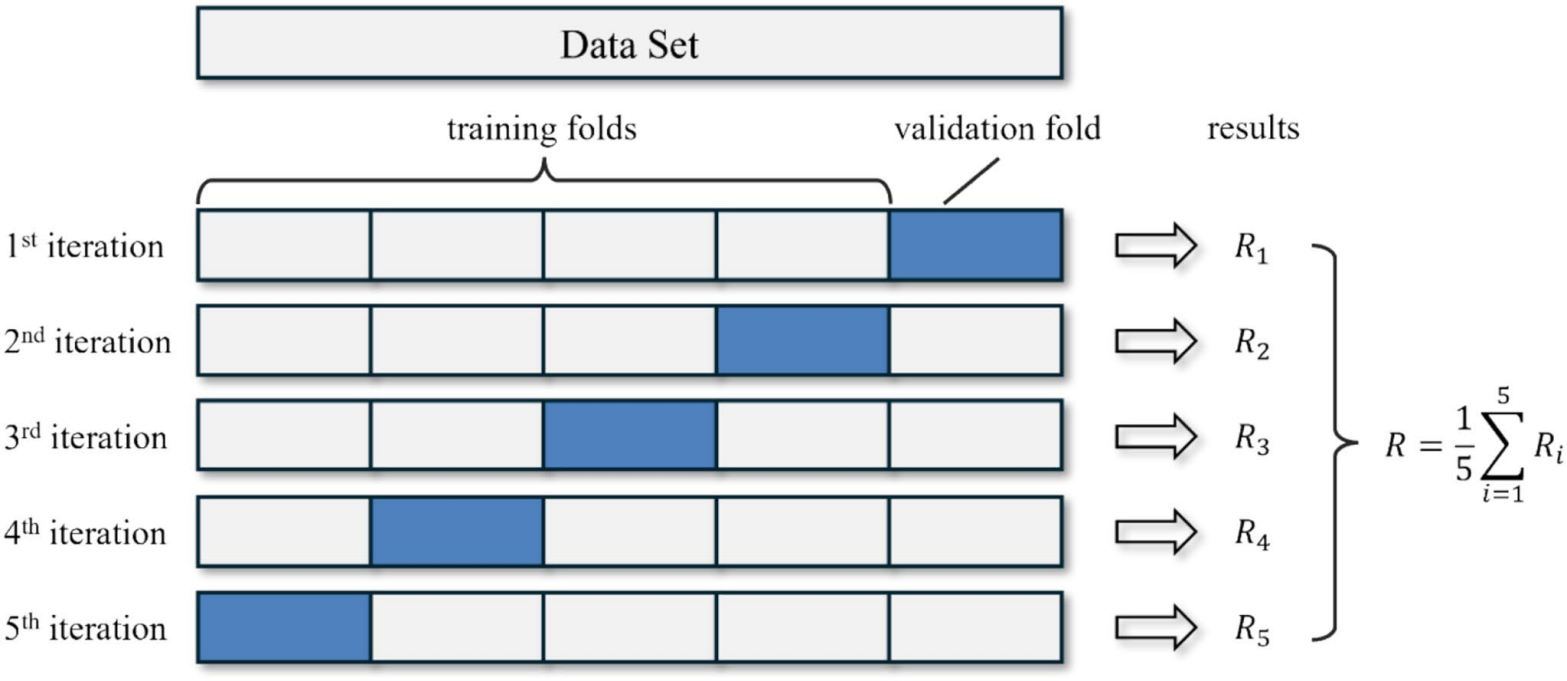
Illustration of 5-fold validation. Diagram showing the data partitioning strategy for model validation. The dataset (*N* = 205) was divided into five equal subsets. In each iteration, four subsets (80%) were used for training and one subset (20%) for validation. This process was repeated five times to ensure all data were used for both training and validation. Final performance metrics were calculated as the average across all folds.

The validation process employed in this study was centered around a 5-fold cross-validation strategy, a robust method for assessing the predictive model’s performance while mitigating overfitting risks. Specifically, the dataset was divided into five parts, each representing 20% of the total data, enabling us to employ a K-fold cross-validation with K = 5. As shown in Figure 4, this strategy entailed using 4 out of the 5 partitions for training, while the remaining one-fifth was reserved for validation in each iteration. The process iterates five times, allowing each fold to serve as the validation set once. After completing the five iterations, we calculated the averaged results obtained from each iteration to derive the final experimental outcome. This averaging process yielded a stable and reliable performance indicator by mitigating the potential impact of variability or bias from any singular data split. Consequently, the averaged results provided a comprehensive evaluation of the model’s predictive capabilities, supporting further optimization efforts based on solid evidence.

The primary advantage of this approach was its ability to provide a comprehensive evaluation of the model’s performance across different data splits. By ensuring that every data point was utilized for both training and validation across different iterations, this method yielded more reliable and generalizable performance metrics. Importantly, it captured the variability and potential biases across different subsets, offering a balanced assessment. Moreover, five-fold cross-validation reduced the problems associated with single train-test splits by allowing all samples to be used for both training and evaluation. This method significantly minimizes variance in performance evaluation, thus providing a more accurate reflection of the model’s ability to generalize to unseen data.

### 5.3 Evaluation metrics

In assessing the diagnostic and prognostic abilities of ACC, our predictive outputs encompassed both categorical and numerical variables. To accurately evaluate the model’s performance, it was essential to employ appropriate metrics for each type of variable. Here, we delineate the evaluation criteria for each variable type, ensuring a robust analysis of model effectiveness. The overall confusion matrix is presented in Table 2.

**Table 2:** The confusion matrix.

Actual/Predicted	Actual positive (1)	Actual negative (0)
Predicted positive (1)	TP	FP
Predicted negative (0)	FN	TN

Standard 2 × 2 confusion matrix defining true positives (TP), false positives (FP), true negatives (TN), and false negatives (FN) used for calculating precision, recall, F1-score, and AUC.

### 5.4 Categorical prediction

For categorical variables, the model was used to classify outcomes into discrete categories, leading to four possible scenarios:

True Positives (TP): Patients who have the disease and are correctly predicted by the model as having the disease.
False Positives (FP): Patients who do not have the disease but are incorrectly predicted as having the disease by the model.
True Negatives (TN): Patients who do not have the disease and are correctly predicted by the model as not having the disease.
False Negatives (FN): Patients who have the disease but are incorrectly predicted by the model as not having the disease.

Based on the four possible cases, we utilized four commonly used evaluation metrics to assess the model’s performance: precision, recall, F1-score, and AUC.

Precision: This metric measures the proportion of true positive predictions among all positive predictions made by the model. It reflects the accuracy of positive predictions.


$$\mathrm{Precision}=\frac{\mathrm{TP}}{\mathrm{TP}+\mathrm{FP}}$$

Recall: Also known as sensitivity, recall evaluates the model’s ability to identify all actual positive cases. It is the ratio of true positives to the total number of actual positive instances.


$$\mathrm{Recall}=\frac{\mathrm{TP}}{\mathrm{TP}+\mathrm{FN}}$$

F1-Score: The F1-score is the harmonic mean of precision and recall, providing a single metric that balances both false positives and false negatives.


$$F1-\mathrm{Score}=2\times \frac{\mathrm{Precision}\times \mathrm{Recall}}{\mathrm{Precision}+\mathrm{Recall}}$$

AUC: The AUC is derived from the ROC curve and represents the model’s ability to distinguish between different classes. A higher AUC indicates better model performance in class separation.

These metrics collectively offered comprehensive insights into the model’s categorical prediction performance across various dimensions.

### 5.5 Numerical prediction

For numerical variables, which entailed predicting numerical prediction outcomes, we utilize Mean Absolute Error (MAE), Mean Squared Error (MSE) and R-squared ( $R^{2}$ ) to assess the model’s performance.

MAE: MAE measures the average magnitude of errors in predictions, without considering their direction. It provides a straightforward indication of model accuracy by assessing the average difference between predicted and actual values.


$$\mathrm{MAE}=\frac{1}{N}\overset{N}{\underset{i=1}{\sum }}\left|{\hat{y}}_{i}-y_{i}\right|$$

MSE: MSE evaluates the average squared difference between predicted and actual values. By squaring the errors, MSE penalizes larger discrepancies more heavily, emphasizing the importance of reducing significant prediction errors.


$$\mathrm{MSE}=\frac{1}{N}\overset{N}{\underset{i=1}{\sum }}{\left({\hat{y}}_{i}-y_{i}\right)}^{2}$$

$R^{2}$ : $R^{2}$ represents the proportion of variance in the dependent variable that is predictable from the independent variables. Values closer to 1 indicate that a substantial proportion of variance is captured by the model, reflecting a strong predictive capability.


$$R^{2}=1-\frac{\overset{N}{\underset{i=1}{\sum }}{\left({\hat{y}}_{i}-y_{i}\right)}^{2}}{\overset{N}{\underset{i=1}{\sum }}{\left(\bar{y}-y_{i}\right)}^{2}},\mathrm{where}\;\bar{y}=\frac{1}{N}\overset{N}{\underset{i=1}{\sum }}y_{i}$$

By utilizing these evaluation metrics, we can comprehensively assess and refine our predictive models for ACC, ensuring reliable diagnostic and prognostic outcomes across both categorical and numerical prediction tasks.

## 6 Results

### 6.1 Main experiments

We conducted extensive experiments on the collected dataset. The experimental results for categorical and numerical predictions are presented in Tables 3, 4, respectively. As noted in Methods, the categorical outcomes represent domain-specific impairments, while the numerical outcomes (Gesell DQ and Gross Motor Score) reflect global developmental status.

**Table 3:** Experimental results of categorical prediction.

Categorical prediction	Precision	Recall	F1-Score	AUC
Intellectual disability	0.99	0.99	0.99	0.99
Communication disorder	0.98	0.98	0.98	0.98
ASD	1.00	1.00	1.00	1.00
Specific learning disorder	1.00	1.00	1.00	1.00
ADHD	1.00	1.00	1.00	1.00
Tic disorder	1.00	1.00	1.00	1.00
Cerebral palsy	0.75	0.72	0.72	0.74
Genetic abnormality	0.98	0.98	0.98	0.99
Epilepsy	0.95	0.95	0.94	0.67
Abnormal EEG	0.98	0.98	0.98	0.98
Developmental coordination disorder	1.00	1.00	1.00	1.00
Global developmental delay	0.98	0.98	0.98	0.99

Performance metrics for 12 neurodevelopmental disorders using 5-fold cross-validation. AUC ≥ 0.98 for most conditions; moderate performance for cerebral palsy (AUC = 0.74) and epilepsy (AUC = 0.67). ASD, autism spectrum disorder; ADHD, attention-deficit/hyperactivity disorder; DCD, developmental coordination disorder; GDD, global developmental delay; SLD, specific learning disorder; EEG, electroencephalogram.

**Table 4:** Experimental results of numerical prediction for developmental scores.

Numerical prediction	MAE ↓	MSE ↓	$R^{2}$
Gesell score	0.10	0.02	0.62
Gross motor score	0.10	0.01	0.63

Performance metrics for Gesell score and Gross Motor Function Measure (GMFM) using 5-fold cross-validation. ↓ indicates lower values are better. MAE, mean absolute error; MSE, mean squared error.

From Table 3, we observed that the model demonstrated an overall high level of accuracy in categorical prediction. For most conditions, the results indicate strong predictive performance, with the model consistently maintaining high discriminative power across the different classes. However, the results also revealed some areas requiring further refinement, particularly concerning the moderate performance observed in cerebral palsy and epilepsy.

From Table 4, it was observed that the model’s performance in numerical prediction demonstrated a consistent level of accuracy across both the Gesell Score and the Gross Motor Score, with both scores showing similar performance in terms of MAE (0.10) and MSE (0.02 for Gesell Score and 0.01 for Gross Motor Score), indicating that the model minimized large prediction errors effectively. However, the $R^{2}$ results (0.62 for Gesell Score, 0.63 for Gross Motor Score) revealed that the model explained approximately 62–63% of the variance in these scores, leaving 37–38% of the variability unaccounted for. This indicated that while the model performed reasonably well, there were likely additional factors or complexities in the data that are not fully captured by the current model.

### 6.2 SHAP-based feature contribution analysis

To further enhance the interpretability of the prediction model, we conducted SHAP analysis to quantify the contribution of individual features to the prediction of the Gesell Score (Lundberg and Lee, 2017). This approach enabled a comprehensive understanding of how input variables influenced the predicted developmental outcomes.

**Figure 5:**
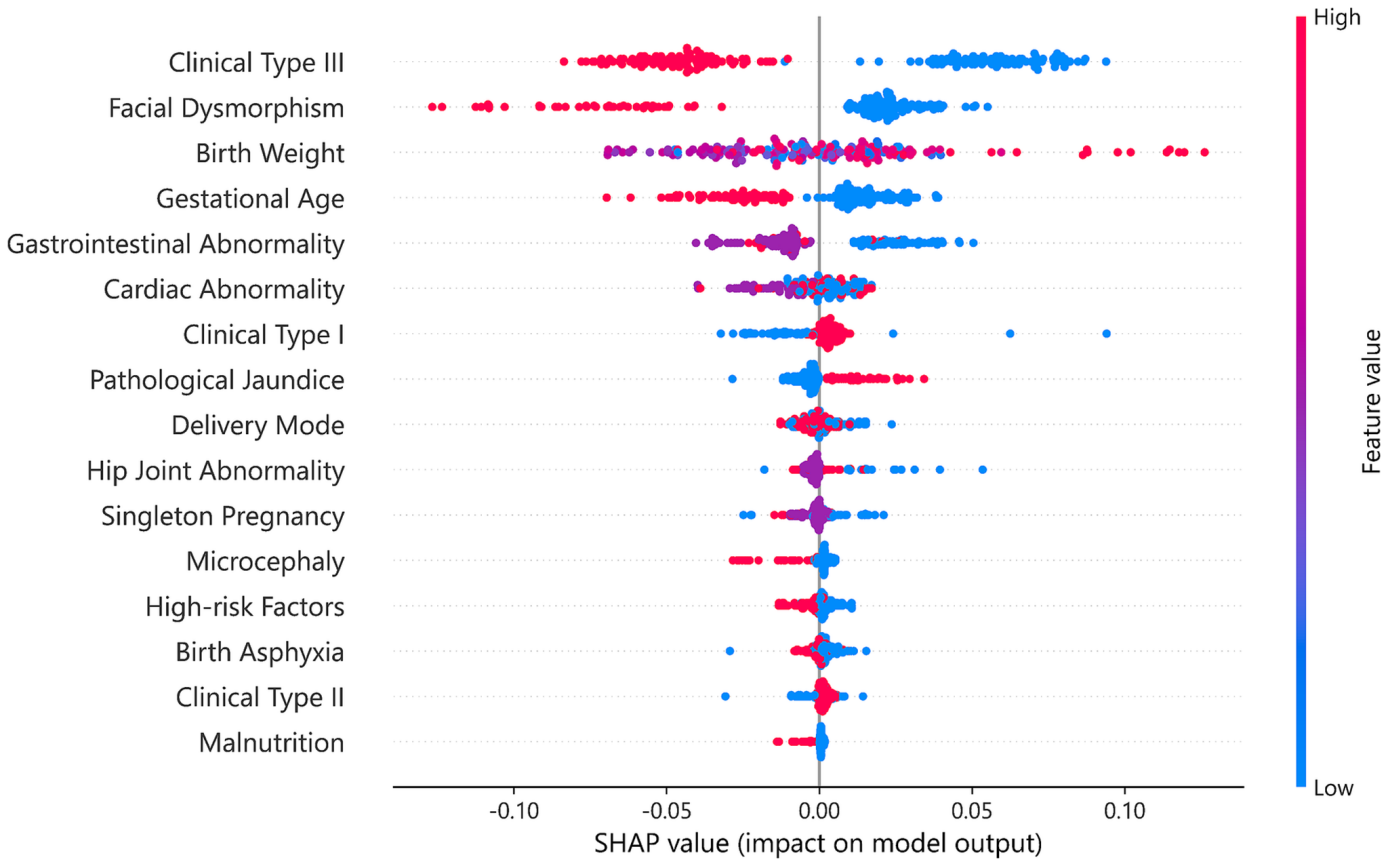
SHAP summary plot illustrating the contribution of each input feature to the prediction of Gesell score. SHAP (Shapley Additive Explanations) analysis showing the top 10 features influencing developmental outcome prediction. Each dot represents a patient, with color indicating feature value (red: high, blue: low) and horizontal position indicating SHA*p* value (impact on model output). Clinical type III (extracranial malformation) showed the strongest negative impact, while birth weight demonstrated a positive contribution to developmental outcomes.

As illustrated in Figure 5, Clinical Type III, representing the presence of extracranial malformations, was the most influential feature, with a strong negative impact on developmental outcomes. This suggested that patients with extracranial abnormalities tended to receive lower predicted Gesell Scores, which was consistent with their known association with increased developmental risk. Facial Dysmorphism also showed a significant negative contribution, reflecting its association with underlying genetic or syndromic conditions. Among numerical variables, Birth Weight contributed positively to the prediction, indicating that higher birth weight was linked to better developmental outcomes. In contrast, Clinical Type II, indicating the presence of other intracranial malformations beyond ACC, and Malnutrition were associated with lower predicted scores, underscoring the adverse effects of systemic congenital conditions.

Overall, the SHAP analysis offered a clear and interpretable understanding of how the model integrated clinical features to predict developmental outcomes. It highlighted the key roles of Clinical Type III, Facial Dysmorphism, and Birth Weight, while demonstrating the model’s capacity to capture complex feature interactions. These insights improved model transparency and provided useful guidance for identifying high-risk cases and informing early intervention strategies.

### 6.3 Comparative analysis with traditional machine learning methods

**Figure 6:**
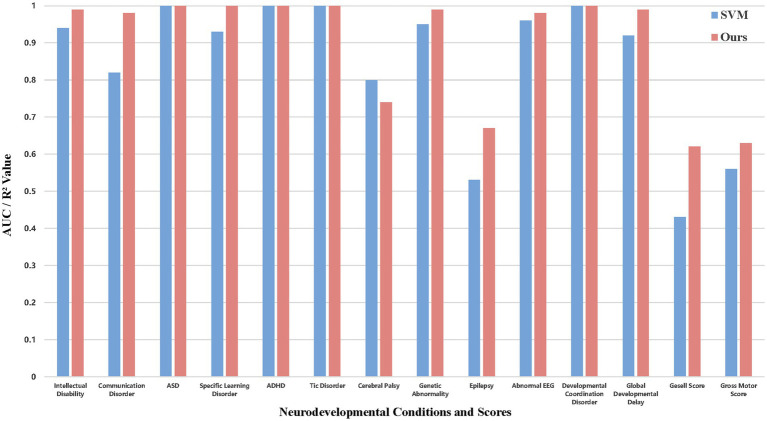
Performance comparison between DNN and SVM models. Bar charts comparing area under the receiver operating characteristic curve (AUC) for 12 categorical prediction tasks and coefficient of determination (R^2^) for 2 numerical prediction tasks. The DNN model (dark bars) outperformed the support vector machine (SVM) baseline (light bars) in most tasks, with notable improvements in Communication Disorder (AUC: 0.98 vs. 0.82) and Gesell Score (R^2^: 0.62 vs. 0.43). Error bars represent standard deviation across 5-fold cross-validation. *p* < 0.001 for DNN vs. SVM comparison. ASD, autism spectrum disorder; ADHD, attention-deficit/hyperactivity disorder; CP, cerebral palsy; DCD, developmental coordination disorder; GDD, global developmental delay; SLD, specific learning disorder.

To further validate the effectiveness of our proposed deep neural network (DNN)-based predictive model, we conducted a comparative analysis with a widely used traditional machine learning method, Support Vector Machine (SVM) (Cortes and Vapnik, 1995; Gallifant et al., 2025). The comparative results are illustrated in Figure 6, which presents AUC for categorical prediction tasks and $R^{2}$ for numerical prediction tasks across both models.

As shown in Figure 6, our model consistently outperformed SVM in the majority of the prediction tasks. Significantly, for predictions such as Communication Disorder and Gesell Score, our model achieved significant performance gains, with AUC increasing from 0.82 to 0.98, and $R^{2}$ improving from 0.43 to 0.62.

These improvements highlighted the DNN model’s superior ability to capture complex, non-linear relationships inherent in high-dimensional clinical data. However, it is worth noting that for Cerebral Palsy, our model slightly underperformed compared to SVM (AUC of 0.74 vs. 0.80). This suggested that for certain conditions with potentially less complex or more structured data distributions, traditional machine learning methods like SVM may still retain some advantages in generalization.

Overall, the comparative analysis confirmed that our proposed DNN-based framework offered significant advantages over traditional machine learning methods, especially in handling complex, heterogeneous datasets typical in ACC diagnosis and prognosis. The ability to model non-linear interactions, combined with robust feature extraction capabilities, positioned our approach as a more reliable and accurate solution for clinical decision support.

### 6.4 Ablation study

**Figure 7:**
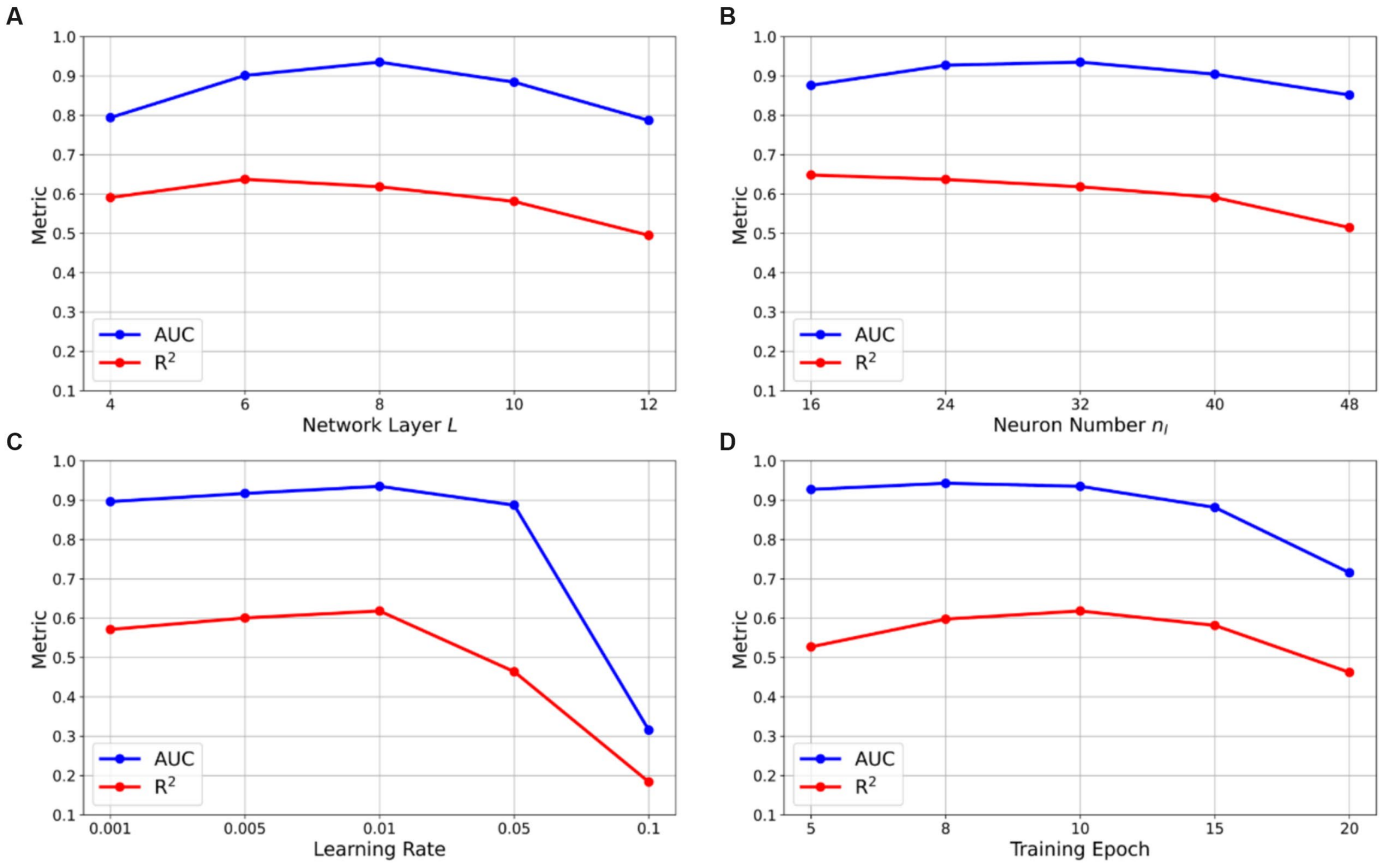
Ablation study of hyperparameters with respect to AUC and $R^{2}$ . Line graphs showing the effect of hyperparameter variations on model performance: **(A)** number of hidden layers, **(B)** number of neurons per layer, **(C)** learning rate, and **(D)** training epochs. Performance was evaluated using mean AUC for categorical variables and mean R^2^ for numerical variables. Optimal configuration (indicated by arrows) was determined as 8 hidden layers, 16 neurons per layer, a learning rate of 0.01, and 10 training epochs. Deviations from these values resulted in decreased performance due to underfitting or overfitting. Shaded areas represent standard deviation across cross-validation folds.

Our predictive model was trained with several key hyperparameters, including the number of network layers L , the number of neurons in each hidden layer $n_{l}$ , the learning rate, and the training epochs. To determine the optimal configuration, we compared model performance across different parameter settings. In the ablation study, each hyperparameter was varied individually while keeping the others constant. To illustrate the impact of these hyperparameters on the model’s performance, we employed the average AUC for categorical predictive variables and the average $R^{2}$ for numerical predictive variables as key indicators. This approach allowed us to effectively assess how each hyperparameter variation influenced the model’s ability to classify and predict outcomes. The results of the ablation study are presented in Figure 7.

Based on the ablation experiments, the hyperparameter configurations were tuned to achieve optimal results with the number of network layers L=8 , the number of neurons in each hidden layer $n_{l}=16$ , a learning rate of 0.01, and a training epoch of 10. It is noteworthy that an excessive number of network layers and neurons can substantially increase the model’s parameter count, which was challenging to train given our limited dataset, often leading to overfitting. Additionally, when the learning rate was excessively high, such as 0.1, the model’s performance severely deteriorated due to the difficulties in convergence. Moreover, too many training epochs could also cause overfitting, further impacting the model’s performance negatively.

## 7 Discussion

This study constructed and externally validated a predictive model integrating structured cranial MRI labels with multidimensional clinical data to assess neurodevelopmental outcomes in children with agenesis of ACC. Through systematic comparison and optimization of image encoding methods, model architecture, and category imbalance mitigation strategies, a stable final model was obtained. The model demonstrated outstanding performance across 12 core developmental phenotype categorical prediction tasks (mean ROC-AUC: 0.97; balanced accuracy: 88.3%). For numerical prediction such as Gesell developmental scores and gross motor function ratings, it demonstrated low prediction error (MAE = 0.10) and high variance explanation (R^2^ ≥ 0.62),significantly outperforming benchmark models based on traditional machine learning (e.g., support vector machines). The model maintained stability across different clinical subgroups. To enhance clinical credibility, we employed the SHAP framework for model interpretation, identifying and quantifying key predictive features and their directional contributions, thereby increasing transparency in the decision-making process.

In recent years, deep learning has been widely applied in the study of neurodevelopmental disorders, particularly excelling at handling high-dimensional heterogeneous data (Beyreli et al., 2022). This study compared the effects of different network architectures and initialization strategies, finding that fully connected networks of appropriate depth combined with ReLU activation functions generally outperformed support vector machines (SVMs) in capturing nonlinear associations, with an average AUC improvement of 7.4–11.2%. However, SVMs demonstrated slightly superior performance in specific prediction tasks such as cerebral palsy, suggesting traditional algorithms retain value when feature-outcome relationships are near-linear or data distributions are balanced. Future research may explore hybrid modeling strategies that dynamically select algorithms based on task characteristics to balance predictive accuracy and robustness.

In terms of image processing, the current model incorporates only structured labels based on MRI reports and does not utilize raw image pixel data. While this approach enhances computational and training efficiency, it may overlook subtle details such as cortical thickness, white matter volume, and diffusion metrics. With the advancement of multi-center image sharing platforms, future approaches could directly feed high-resolution 3D images into convolutional neural networks and perform end-to-end fusion learning with clinical variables. This is expected to further enhance the diagnostic efficacy for complex phenotypes like cerebral palsy and epilepsy.

In terms of clinical features, the SHAP plot in Figure 5 shows that Clinical Type III exerts the strongest negative influence, indicating that the presence of extracranial malformations substantially increases developmental risk, in line with established clinical understanding (Andrews et al., 2009). Facial Dysmorphism also contributes negatively, reflecting its association with underlying genetic or syndromic conditions that affect neurodevelopment. Birth Weight shows a positive effect, underscoring its role as a protective perinatal factor, as it potentially reflects both gestational maturity and adequacy of intrauterine growth. In contrast, Clinical Type II and Malnutrition exhibit negative contributions, highlighting the adverse impact of complex intracranial abnormalities and systemic congenital conditions. Together, these findings enhance model transparency, support clinical interpretability, and provide practical guidance for identifying high-risk subgroups that may benefit from early intervention (Oberman et al., 2016; Dong and Greenough, 2004; Pastura and Land, 2017).

To address the common issue of data imbalance in rare disease research, we employed a weighted cross-entropy loss function combined with a multi-scale under sampling strategy. This approach elevated the F1-score for the minority class from 0.51 to 0.78 without significantly compromising overall accuracy. Furthermore, by employing appropriate interpolation methods to address missing data, we enhanced dataset integrity and model robustness, providing a scalable analytical framework for conducting multicenter studies on rare malformations.

Regarding interpretability methods, combining SHAP with ablation experiments not only validated the importance of key features but also indicated that certain variables contributed less significantly, such as mode of delivery and some high-risk factors, providing a reference for subsequent feature selection. It should be noted that as a local interpretation method, SHAP values may fluctuate with background samples. Therefore, resampling techniques like Bootstrap should be employed to assess their stability. For continuous outcomes, future studies may concurrently utilize methods such as LIME or ALE to provide more intuitive decomposition of error sources (Zhou et al., 2025).

### 7.1 Limitations

This study has certain limitations. First, MRI examinations were performed at varying ages across the cohort. The timing of the scan relative to brain maturation may influence the detection of certain abnormalities (e.g., cortical migration disorders), which could affect the consistency of imaging features and their predictive power for outcomes like epilepsy. Second, this single-center retrospective design lacked external validation for racial diversity and imaging protocol variability. Third, genetic information was limited to categorical prediction labels, without integration of whole-exome or structural variant data. Finally, dynamic variables such as rehabilitation training and household socioeconomic status were not collected, potentially limiting the explanation of the remaining 37–38% variance.

## Data Availability

The raw data supporting the conclusions of this article will be made available by the authors, without undue reservation, to any qualified researcher upon reasonable request. The data are not publicly available due to privacy restrictions related to patient medical records and institutional policies protecting sensitive health information of minors.
